# Event-Triggered State Filter Estimation for INS/DVL Integrated Navigation with Correlated Noise and Outliers

**DOI:** 10.3390/s25051545

**Published:** 2025-03-02

**Authors:** Xiaolei Ma, Zhengrong Wei, Weicheng Liu, Shengli Wang

**Affiliations:** College of Ocean Science and Engineering, Shandong University of Science and Technology, Qingdao 266590, China; 15966134015@163.com (X.M.); 15266287097@163.com (W.L.); shlwang@sdust.edu.cn (S.W.)

**Keywords:** autonomous underwater vehicles, inertial navigation system/doppler velocity log integrated navigation, event-triggered mechanism, correlation noise filter

## Abstract

The Inertial Navigation System (INS) and Doppler Velocity Log (DVL) combination navigation system has been widely used in Autonomous Underwater Vehicles (AUVs) due to its independence, stealth, and high accuracy. Compared to the standalone INS or DVL, the integrated system provides continuous and accurate navigation information. However, the underwater environment is complex, and system noise and observation noise may not satisfy the condition of mutual independence. In addition, the DVL may produce abnormal measurement values during operation. In this study, an Event-Triggered Correlation Noise Filter (ETCNF) method was designed for fusing INS and DVL data. An auxiliary matrix was introduced to decouple the correlated noise, allowing novel state estimation. Moreover, the event-triggered mechanism detected and eliminated abnormal values in DVL measurements, which improved the positioning accuracy and robustness of the INS/DVL integrated system. Finally, simulation experiments were conducted to verify the effectiveness and superiority of the proposed algorithm.

## 1. Introduction

As various countries are paying more and more attention to marine resources and rights, the development of AUVs has attracted considerable attention, and their underwater applications have become increasingly pervasive. AUVs are employed for a wide array of missions, including ocean surveys, mine clearance, and data collection in both marine and riverine environments. Precise positioning and navigation are critical for ensuring the accuracy of the data collected in various applications [1].

For AUVs, a Global Positioning System (GPS) is typically suitable for surface operations, including AUV deployment, AUV recovery, and the repositioning of AUVs on the surface. However, the rapid attenuation of GPS signals underwater renders it unusable for AUV missions taking place underwater [2]. The intricate and dynamic marine environment further complicates the task of precise navigation and positioning for AUVs [3]. Without underwater navigation technology, AUVs are unable to accomplish their predetermined tasks. In contrast to aerial navigation, underwater navigation is distinguished by extended operational durations, constrained information sources, and a high degree of concealment [4]. Underwater navigation technology can provide AUVs with accurate positional, velocity, and attitude information.

The common underwater navigation methods primarily include the use of an INS, acoustic navigation, and geophysical navigation [5]. An INS offers such advantages as independence, concealment, and real-time performance and is frequently used as the primary navigation system. However, INSs also face challenges, notably the accumulation of errors over time. Acoustic navigation mainly involves the use of a DVL, a Long Baseline (LBL), a Short Baseline (SBL), and an Ultrashort Baseline (USBL). A DVL is an active sonar system that leverages the Doppler effect to measure velocity relative to the seabed or water column, providing high-precision velocity information for the carrier. However, the marine environment is exceedingly complex. Ocean current interference, marine geological conditions, and fish-school effects may cause anomalies or even disruptions in DVL performance [6]. LBLs, SBLs, and USBLs differ in baseline length, sound wave frequency, volume, and positioning accuracy [7,8]. Given the low energy loss of sound waves in water, they are important for underwater communication and positioning [9,10,11]. However, acoustic navigation systems are susceptible to interference (including underwater background noise as well as noise from engines and equipment) as well as the influences of physical obstacles and complex propagation phenomena (e.g., multipath reception, echoes, refraction in water layers, and reverberation), particularly over extended distances [12]. These limitations restrict their application in situations demanding high positioning accuracy. Geophysical field-matching navigation furnishes AUVs with positioning information by aligning real-time-measured physical characteristic data with pre-stored physical field map data within the system [13]. Hence, geophysical field matching provides high navigation and positioning accuracy. However, it necessitates establishing a geophysical field database for the operational sea area in advance, whereas creating a global sea area database is challenging and costly [14]. The positioning of AUVs is rarely achievable with a single navigation method alone. Instead, the integration of multiple navigation techniques can complement their strengths, thereby enabling accurate AUV positioning.

INS/DVL integrated navigation is one of the main navigation techniques for AUVs [15]. The information fusion method can directly affect the positioning accuracy of INS/DVL integrated navigation. The Kalman filter (KF) is known to be a very useful processing method. Reference [16] performed basic combination and integration of the INS/DVL integrated navigation system using the extended Kalman filter (EKF) technique. Reference [17] used two different filters, the KEF and untracked Kalman filter (UKF), to fuse INS and DVL data. The experimental results showed that position derivation requires the quadratic integration of the measured acceleration, resulting in the EKF result being closer to the real path of the vehicle than that of the UKF. Increasing the number of sigma points may improve the accuracy of UKF. Reference [18] proposed an adaptive Kalman filter (AKF) with a recursive noise estimator based on maximum a posteriori estimation and one-step smoothing filtering. This method allows the estimation of the process states and noise parameters when the process and measurement noise statistics are unknown or time-varying. Reference [19] proposed an SINS/DVL integrated navigation and positioning method based on improved adaptive filtering technology. The navigation parameters of the system were filtered using an adaptive gain filtering method. Reference [20] proposed a new robust adaptive federated strong tracking Kalman filter (RAFSTKF) algorithm for INS/DVL data fusion that was robust to the uncertainty of the system model. Reference [21] introduced a forgetting factor and a variable sliding window on the basis of the Sage-Husa adaptive Kalman filtering algorithm (SHAKF), further enhancing the robustness and adaptability of the adaptive filter. Reference [22] proposed a robust interactive multi-model (RIMM) algorithm with an adaptive model set, which had good accuracy and robustness in a heavy-tail non-Gaussian noise environment.

Due to the intricacy of the underwater environment, some previously proposed filtering methods often overlook the correlation between system noise and observation noise in the INS/DVL integration process. References [23,24] took this correlation into account when fusing INS with partial DVL measurements. By considering the cross-covariance matrix of correlated processes and measurement noise, partial DVL measurements were combined with additional information (e.g., water-penetrating navigation solutions), yielding calculated velocity measurements that augment the INS. Reference [25] introduced a method for calculating the cross-covariance matrix of process and measurement noise in a discrete-time Kalman filter. However, the correlation between system noise and observation noise occurred at different instances rather than simultaneously. Reference [26] proposed an angular rate-assisted rapid initial alignment method based on robust Kalman filtering. In this method, a state augmentation approach is employed to address the correlation between process noise and measurement noise in angular rate-assisted methods. Unfortunately, the dimensionality of the filter is higher, resulting in a substantial increase in computational complexity. Although there is a lack of research on correlative noise in underwater integrated navigation applications, in the field of nonlinear system state estimation with correlated noise, there are many studies worthy of reference. Reference [27] proposed a Gaussian approximation recursive filter (GASF) for a class of nonlinear stochastic systems for cases where the process and measurement noises are correlated with each other. Through presenting the Gaussian approximations pertaining to the two-step state posterior predictive probability density function (PDF) and the one-step measurement posterior predictive PDF, a general GASF framework in the minimum mean square error (MMSE)-sense was derived. Reference [28] studied the filtering problem concerning nonlinear systems with cross-correlation between process and observation noise in the same period. By introducing a decorrelation scheme and reconstructing the pseudo nonlinear process equation, the pseudo process noise and observation noise were no longer cross-correlated, and the unscented Kalman filter (UKF) was used to filter the nonlinear problems. Reference [29], in studying Kalman filtering for linear systems where the same period process and observed noise are correlated, derived an alternative formula for the filter; namely, conditional process noise was used in the prediction step instead of the original noise.

When influenced by ocean currents and marine organisms, DVL observations may exhibit abnormal values. The elimination of such anomalies is vital for ensuring the positioning accuracy of INS/DVL integrated navigation [30,31]. Reference [32] presented a simple method for detecting and getting rid of abnormal values in underwater operations. It involves utilizing a simple chi-square statistical test to evaluate each new measurement, discarding those that produce significant residuals compared to the values predicted by the Extended Kalman Filter (EKF). Yet, abnormal values may be overlooked if the test threshold is set too high, and misjudgment may occur if it is set too low. Reference [33] describes an adaptive Kalman filter for suppressing abnormal values, where the presence of anomalies is identified based on the magnitude of the residuals. The threshold value for this method is related to system noise, underwater conditions, and measurement equipment noise. In applications, this value needs to be adjusted according to the actual measured data. The extension of the concept of Huber’s M-estimation methodology to the problem of robust Kalman filtering has been widely researched and emphasized to a greater extent than other approaches since it is informed by maximum likelihood estimation, which makes it more natural and rather simple [34,35]. Reference [36] focused on deriving a robust derivative-free KF using Huber’s M-estimation methodology without linearization or statistical linearization. This method can handle contaminated Gaussian noise and outliers in measurements. References [37,38] introduced Huber’s M-estimation for robust estimation in underwater integrated navigation. Huber’s M-estimation was utilized to rectify the measurement residual sequence and reconstruct measurement information, thereby addressing the issue of reduced filtering precision in the presence of outliers. Nevertheless, the optimization of Huber’s M-estimation involves iterative calculations, increasing the complexity of a model. Reference [39] presented a Long Short-Term Memory Extended Exponentially Weighted Kalman Filter (LSTM-EEWKF) algorithm assisted by Long Short-Term Memory (LSTM) neural networks. When DVL measurements are contaminated by outliers, the trained LSTM neural network provides correction information for the system. The main disadvantages of this method are that it requires sufficient training data and the training is time-consuming.

In this study, an ETCNF algorithm was proposed on account of the correlation between system noise and observation noise caused by the complexity of underwater environments. An auxiliary matrix was added to decouple system noise and observation noise, achieving new state prediction and consequently improving navigation and positioning accuracy. Furthermore, in multi-sensor information fusion systems, in order to maintain the stability of estimation accuracy and reduce energy consumption at the same time, an event-triggered mechanism is often used to control the rate at which data are transmitted from the sensor to the fusion center [40,41]. Inspired by this, an event-triggered mechanism was introduced to detect and eliminate abnormal values in DVL measurements, enhancing the robustness of this system.

The main contributions of this study are outlined as follows.

(1) An ETCNF algorithm was developed. An auxiliary matrix was added to de-correlate system noise and observation noise. A novel INS/DVL integrated navigation filtering model was established, effectively addressing the problematic correlation that existed between system noise and observation noise occurring at the same time.
(2) An event-triggered mechanism was employed to effectively detect and eliminate abnormal values in DVL measurements, thereby preventing reductions in filtering precision due to such abnormal values.
(3) Rigorous simulation experiments were conducted, verifying that the proposed integrated navigation method was effective.

The rest of this article is organized as follows. The INS/DVL navigation model is described in Section 2. In the third Section, the ETCNF method is presented. The simulation results are shown in Section 4. See Section 5 for the conclusions.

## 2. INS/DVL Integrated Navigation System Model

**Figure 1:**
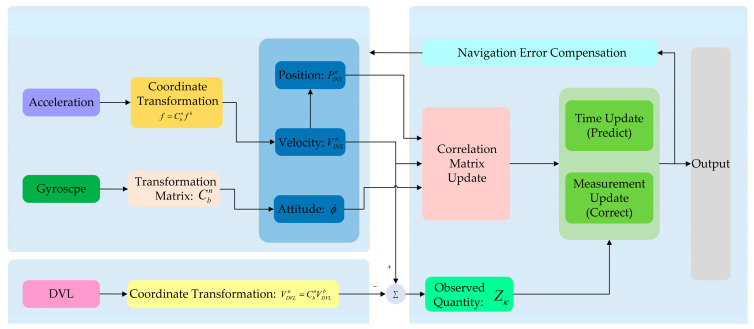
Diagram of the loose combination structure of the INS/DVL integrated navigation system.

In the INS/DVL integrated navigation system, the INS acquires information on attitude, speed, and position by using a gyroscope and an accelerometer, while the DVL provides velocity observation information. The above information is fused using a Kalman filter, and the estimated results are obtained for correcting the INS to obtain more accurate navigation information. The loose combination structure of a common INS/DVL integrated navigation system is shown in Figure 1.

### 2.1. System Error State Model

After the calibration of the DVL, the calibration factor error and installation deviation angle are not considered. The state variable X is used to select the 15-dimensional state error of INS, expressed as

(1) $$X={\left[\phi^{T}\;{\left(\delta V^{n}\right)}^{T}\;(\delta r{)}^{T}\;{\left(\varepsilon^{b}\right)}^{T}\;{\left(\nabla^{b}\right)}^{T}\right]}^{T},$$

where $\phi^{T}$ represents the misalignment angles in three directions; $\delta V^{n}$ represents the velocity error vector in E-N-U directions in the n system coordinate system; δr represents the position error vector; $\varepsilon^{b}$ represents the gyroscope drift vector in a right-front-up orientation in the b coordinate system; and $\nabla^{b}$ represents the accelerometer bias vector in the right-front-up orientation in the b coordinate system.

The state equation for the integrated navigation system is

(2) $${\dot{X}}_{\kappa +1}=F_{\kappa }X_{\kappa }+W_{\kappa }$$

And the system state transition matrix $F_{\kappa }$ can be written as follows:

(3) $$F_{k}=\left[\begin{array}{ccccc} F_{\phi \phi } & F_{\phi v} & F_{\phi r} & -C_{b}^{n} & 0_{3\times 3}\\ F_{v\phi } & F_{vv} & F_{vr} & 0_{3\times 3} & C_{b}^{n}\\ 0_{3\times 3} & F_{rv} & F_{rr} & 0_{3\times 3} & 0_{3\times 3}\\ & & 0_{6\times 15} & & \end{array}\right]$$

where $F_{\phi \phi },F_{\phi v},F_{\phi r},F_{v\phi },F_{vv},F_{vr},F_{rv},F_{rr}$ can be expressed as follows:

(4) $$F_{\phi \phi }=\left[\begin{array}{ccc} 0 & \frac{V_{E}^{n}\tan L}{R_{N}+h}+w_{ie}\sin L & -\frac{V_{E}^{n}}{R_{N}+h}-w_{ie}\cos L\\ -\frac{V_{E}^{n}\tan L}{R_{N}+h}-w_{ie}\sin L & 0 & \frac{V_{N}^{n}}{R_{M}+h}\\ \frac{V_{E}^{n}}{R_{N}+h}+w_{ie}\cos L & \frac{V_{N}^{n}}{R_{M}+h} & 0 \end{array}\right]$$

(5) $$F_{\phi v}=\left[\begin{array}{ccc} 0 & -\frac{1}{R_{M}+h} & 0\\ \frac{1}{R_{N}+h} & 0 & 0\\ \frac{\tan L}{R_{N}+h} & 0 & 0 \end{array}\right]$$

(6) $$F_{\phi r}=\left[\begin{array}{ccc} 0 & 0 & \frac{V_{N}^{n}}{{\left(R_{M}+h\right)}^{2}}\\ -w_{ie}\sin L & 0 & -\frac{V_{E}^{n}}{{\left(R_{M}+h\right)}^{2}}\\ \frac{V_{E}^{n}\sec^{2}L}{R_{N}}+w_{ie}\cos L & 0 & -\frac{V_{E}^{n}\tan L}{{\left(R_{M}+h\right)}^{2}} \end{array}\right]$$

(7) $$F_{v\phi }=\left[\begin{array}{ccc} 0 & -f_{U}^{n} & f_{N}^{n}\\ f_{U}^{n} & 0 & -f_{E}^{n}\\ -f_{N}^{n} & f_{E}^{n} & 0 \end{array}\right]$$

(8) $$F_{vv}=\left[\begin{array}{ccc} \frac{V_{N}^{n}\tan L-V_{U}^{n}}{R_{N}+h} & \frac{V_{E}^{n}\tan L}{R_{N}+h}+2w_{ie}\sin L & -\frac{V_{E}^{n}}{R_{N}+h}-2w_{ie}\cos L\\ -\frac{2V_{E}^{n}\tan L}{R_{N}+h}-2w_{ie}\sin L & -\frac{V_{U}^{n}}{R_{M}+h} & -\frac{V_{N}^{n}}{R_{M}+h}\\ \frac{2V_{E}^{n}}{R_{N}+h}+2w_{ie}\cos L & \frac{2V_{N}^{n}}{R_{M}+h} & 0 \end{array}\right]$$

(9) $$F_{vr}=\left[\begin{array}{ccc} \frac{V_{E}^{n}V_{N}^{n}\sec^{2}L}{R_{N}+h}+2w_{ie}(V_{U}^{n}\sin L+V_{N}^{n}\cos L) & 0 & \frac{V_{E}^{n}V_{U}^{n}-V_{E}^{n}V_{N}^{n}\tan L}{{\left(R_{N}+h\right)}^{2}}\\ -\frac{{\left(V_{E}^{n}\right)}^{2}\sec^{2}L}{R_{N}+h}-2w_{ie}V_{E}^{n}\cos L & 0 & \frac{V_{E}^{n}V_{U}^{n}}{{\left(R_{M}+h\right)}^{2}}+\frac{{\left(V_{E}^{n}\right)}^{2}\tan L}{{\left(R_{N}+h\right)}^{2}}\\ -2w_{ie}V_{E}^{n}\sin L & 0 & -\frac{{\left(V_{N}^{n}\right)}^{2}}{{\left(R_{M}+h\right)}^{2}}-\frac{{\left(V_{E}^{n}\right)}^{2}}{{\left(R_{N}+h\right)}^{2}} \end{array}\right]$$

(10) $$F_{rv}=\left[\begin{array}{ccc} 0 & \frac{1}{R_{M}+h} & 0\\ \frac{1}{(R_{M}+h)\cos L} & 0 & 0\\ 0 & 0 & 1 \end{array}\right]$$

(11) $$F_{rr}=\left[\begin{array}{ccc} 0 & 0 & -\frac{V_{N}^{n}}{{\left(R_{M}+h\right)}^{2}}\\ \frac{V_{E}^{n}\tan L}{(R_{M}+h)\cos L} & 0 & -\frac{V_{E}^{n}}{{\left(R_{M}+h\right)}^{2}\cos L}\\ 0 & 0 & 0 \end{array}\right]$$

The process noise matrix $W_{\kappa }$ can be expressed as

(12) $$W_{\kappa }=\left[\begin{array}{c} -C_{b}^{n}w_{g}^{b}\\ C_{b}^{n}w_{a}^{b}\\ 0_{9\times 1} \end{array}\right]$$

where $w_{g}^{b}$ denotes the Gaussian white noise of the gyroscope in coordinate system b; and $w_{a}^{b}$ denotes the Gaussian white noise of the accelerometer in coordinate system b.

### 2.2. Measurement Equation

The DVL can provide velocity information. Therefore, the difference between DVL velocity and INS velocity was selected as the measurement vector, and the following system measurement equation was established:

(13) $$Z_{\kappa }=H_{\kappa }X_{\kappa }+V_{\kappa }$$

where $H_{\kappa }$ represents the measurement transfer matrix; and $V_{\kappa }$ represents measurement noise.

The velocity vector of the DVL can be expressed as

(14) $$\begin{array}{cc} {\tilde{V}}_{DVL}^{n} & ={\tilde{C}}_{b}^{n}C_{d}^{b}V_{DVL}^{d}\\ & =[I-\phi \times ]C_{b}^{n}C_{d}^{b}V_{DVL}^{d}\\ & =V_{DVL}^{n}+V_{DVL}^{n}\times \phi \end{array}$$

where ${\tilde{V}}_{DVL}^{n}$ denotes the velocity vector of DVL with errors; $V_{DVL}^{n}$ and $V_{DVL}^{d}$ is the true value of the DVL in the different coordinate systems.

According to (14), we can obtain the velocity error vector of the DVL as follows:

(15) $$\begin{array}{cc} \delta V_{DVL}^{n} & ={\tilde{V}}_{DVL}^{n}-V_{DVL}^{n}\\ & =V_{DVL}^{n}\times \phi \end{array}$$

Therefore, (13) can be rewritten as

(16) $$\begin{array}{cc} Z_{k} & ={\tilde{V}}_{INS}^{n}-{\tilde{V}}_{DVL}^{n}\\ & =(V_{INS}^{n}+\delta V_{INS}^{n})-(V_{DVL}^{n}+\delta V_{DVL}^{n})\\ & =\delta V_{INS}^{n}-\delta V_{DVL}^{n}\\ & =-V_{DVL}^{n}\times \phi +\delta V_{INS}^{n} \end{array}$$

Then, we can get the measurement transfer matrix $H_{\kappa }$ as follows:

(17) $$H_{k}=[\begin{array}{ccc} -V_{DVL}^{n}\times & I & 0_{3\times 9} \end{array}]$$

## 3. Design of the Proposed Method

To improve the positioning accuracy of the INS/DVL system, we consider the effects of underwater correlated noise and abnormal values in the DVL measurement process on the filtering results, and we propose a new DVL/INS integrated navigation model based on an innovative event-triggered mechanism.

### 3.1. Event-Triggered Mechanism

The advantage of the event-triggered mechanism is that the measurement information from sensors is input for state estimation only if the prerequisite conditions are satisfied. This feature is very conducive to the detection and identification of outliers in DVL measurement. In order to effectively identify outliers, we must introduce the measurement innovation to trigger the event.

Based on (2) and (13), the measurement innovation is

(18) $$\begin{array}{cc} \varepsilon_{\kappa +1} & =E[Z_{\kappa +1}-{\overset{⌢}{Z}}_{\kappa +1|\kappa }|Z_{\kappa }]\\ & =E[Z_{\kappa +1}-H_{\kappa +1}{\overset{⌢}{X}}_{\kappa +1|\kappa }|Z_{\kappa }]\\ & =E[H_{\kappa +1}X_{\kappa +1}+V_{\kappa +1}-H_{\kappa +1}{\overset{⌢}{X}}_{\kappa +1|\kappa }|Z_{\kappa }]\\ & =H_{\kappa +1}{\tilde{X}}_{\kappa +1|\kappa }+V_{\kappa +1} \end{array}$$

We assume the measurement innovation $\varepsilon_{\kappa +1}$ is Gaussian-distributed, with zero mean and the corresponding covariance matrix

(19) $$\begin{array}{cc} Q_{\varepsilon_{\kappa +1}} & =E[\varepsilon_{\kappa +1}\varepsilon_{\kappa +1}^{T}|Z_{\kappa }]\\ & =E[(H_{\kappa +1}{\tilde{X}}_{\kappa +1|\kappa }+V_{\kappa +1}){\left(H_{\kappa +1}{\tilde{X}}_{\kappa +1|\kappa }+V_{\kappa +1}\right)}^{T}|Z_{\kappa }]\\ & =H_{\kappa +1}P_{\kappa +1|\kappa }H_{\kappa +1}^{T}+R_{\kappa +1} \end{array}$$

Since $Q_{\varepsilon_{\kappa +1}}$ is semidefinite, there must be a unitary matrix $U_{\kappa +1}$ that satisfies

(20) $$U_{\kappa +1}Q_{\varepsilon_{\kappa +1}}U_{\kappa +1}^{T}=\Lambda_{\kappa +1}$$

where $\Lambda_{\kappa +1}$ is a diagonal matrix composed of the eigenvalues of $Q_{\varepsilon_{\kappa +1}}$.

Let us define a matrix ${Μ}_{\kappa +1}$:

(21) $$M_{\kappa +1}=U_{\kappa +1}\Lambda_{\kappa +1}^{-\frac{1}{2}}$$

According to (42), it clearly satisfies

(22) $$M_{\kappa +1}M_{\kappa +1}^{T}=Q_{\varepsilon_{\kappa +1}}^{-1}$$

Then, the normalization of $\varepsilon_{\kappa +1}$ is as follows:

(23) $${\bar{\varepsilon }}_{\kappa +1}=M_{\kappa +1}^{T}\varepsilon_{\kappa +1}$$

and ${\bar{\varepsilon }}_{\kappa +1}$ satisfies the standard normal distribution. We define the event-triggered mechanism as follows:

(24) $$\gamma_{\kappa +1}=\left\{\begin{array}{c} 1,\;{\left\|{\bar{\varepsilon }}_{\kappa +1}\right\|}_{\infty }\leq T\\ 0,\;\mathrm{otherwise} \end{array}\right.$$

where T>0 is the threshold for determining whether the measurement is an outlier.

### 3.2. Kalman Filter with Cross-Correlation at the Same Time

Based on Formulas (2) and (13), the initial state is assumed to follow a Gaussian distribution with a mean of ${\overset{⌢}{x}}_{0|0}$ and covariance $P_{0|0}$ which are independent of $W_{\kappa }$ and $V_{\kappa }$. The process noise $W_{\kappa }$ and the measurement noise $V_{\kappa }$ are correlated zero−mean Gaussian white noise satisfying the following:

(25) $$E[W_{\kappa }W_{j}^{T}]=Q_{\kappa }\delta_{\kappa j}$$

(26) $$E[V_{\kappa }V_{j}^{T}]=R_{\kappa }\delta_{\kappa j}$$

(27) $$E[W_{\kappa }V_{j}^{T}]=S_{\kappa }\delta_{\kappa j}$$

where $\delta_{\kappa j}$ is the Kronecker delta function. The final equation demonstrates that $W_{\kappa }$ and $V_{\kappa }$ exhibit cross−correlation solely when the time arguments are the same.

When $W_{\kappa }$ and $V_{\kappa }$ are uncorrelated, we assume that the one-step predictive probability density function $p(X_{\kappa +1}|Z_{\kappa })$ and the filtering probability density function $p(X_{\kappa +1}|Z_{\kappa +1})$ are Gaussian. Based on the fact that $W_{\kappa }$ and $Z_{\kappa }$ are independent, we arrive at

(28) $$E[W_{\kappa }|Z_{\kappa }]=0$$

The filtering process of the INS/DVL integrated navigation system can be obtained as follows:

(29) $${\overset{⌢}{X}}_{\kappa +1|\kappa }=F_{\kappa +!|\kappa }{\overset{⌢}{X}}_{\kappa }$$

(30) $$P_{\kappa +1|\kappa }=F_{\kappa +1|\kappa }P_{\kappa }F_{\kappa +1|\kappa }+Q_{\kappa }$$

(31) $$K_{\kappa +1}=P_{\kappa +1|\kappa }H_{\kappa +1}^{T}{\left(H_{\kappa +1}P_{\kappa +1|\kappa }H_{\kappa +1}^{T}+R_{\kappa +1}\right)}^{-1}$$

(32) $${\overset{⌢}{X}}_{\kappa +1}={\overset{⌢}{X}}_{\kappa +1|\kappa }+K_{\kappa +1}(Z_{\kappa +1}-H_{\kappa +1}{\overset{⌢}{X}}_{\kappa +1|\kappa })$$

(33) $$P_{\kappa +1}=P_{\kappa +1|\kappa }-K_{\kappa +1}H_{\kappa +1}P_{\kappa +1|\kappa }$$

However, we apply the filtering design for the case where $W_{\kappa }$ and $V_{\kappa }$ are correlated, in which case (28) is no longer valid. This further disallows the use of (29)–(33).

To solve the above problems, we introduce an auxiliary matrix $\xi_{\kappa }$. According to Formula (13), we can rewrite (2) as

(34) $$\begin{array}{cc} X_{\kappa +1} & =F_{\kappa }X_{\kappa }+W_{\kappa }+\xi_{\kappa }(Z_{\kappa }-H_{\kappa }X_{\kappa }-V_{\kappa })\\ & =(F_{\kappa }-\xi_{\kappa }H_{\kappa })X_{\kappa }+\xi_{\kappa }Z_{\kappa }+(W_{\kappa }-\xi_{\kappa }V_{\kappa })\\ & =F_{\kappa }^{*}X_{\kappa }+U_{\kappa }+W_{\kappa }^{*} \end{array}$$

where $F_{\kappa }^{*}$, $U_{\kappa }$, and $W_{\kappa }^{*}$ can be expressed as

(35) $$F_{\kappa }^{*}=F_{\kappa }-\xi_{\kappa }H_{\kappa }$$

(36) $$U_{\kappa }=\xi_{\kappa }Z_{\kappa }$$

(37) $$W_{\kappa }^{*}=W_{\kappa }-\xi_{\kappa }V_{\kappa }$$

Our aim is to attain the one-step predictive probability density function $p(X_{\kappa +1}|Z_{\kappa })$ and the filtering probability density function $p(X_{\kappa +1}|Z_{\kappa +1})$. Based on (34) and (25)–(27), $W_{\kappa }^{*}$ and $V_{\kappa }$ must be unrelated; then,

(38) $$E[W_{\kappa }^{*}V_{\kappa }^{T}]=E[(W_{\kappa }-\xi_{\kappa }V_{\kappa })V_{\kappa }^{T}]=S_{\kappa }-\xi_{\kappa }R_{\kappa }=0$$

From the expression above, we can obtain the following:

(39) $$\xi_{\kappa }=S_{\kappa }R_{\kappa }^{-1}$$

And the new covariance of the process noise of (34) is

(40) $$\begin{array}{cc} Q_{\kappa }^{*} & =E[W_{\kappa }^{*}{\left(W_{\kappa }^{*}\right)}^{T}]\\ & =E[(W_{\kappa }-\xi_{\kappa }V_{\kappa }){\left(W_{\kappa }-\xi_{\kappa }V_{\kappa }\right)}^{T}]\\ & =E[W_{\kappa }W_{\kappa }^{T}]-E[W_{\kappa }V_{\kappa }^{T}\xi_{\kappa }^{T}]-E[\xi_{\kappa }V_{\kappa }W_{\kappa }^{T}]+E[\xi_{\kappa }V_{\kappa }V_{\kappa }^{T}\xi_{\kappa }^{T}]\\ & =Q_{\kappa }-S_{\kappa }\xi_{\kappa }^{T}-\xi_{\kappa }S_{\kappa }^{T}+\xi_{\kappa }R_{\kappa }\xi_{\kappa }^{T}\\ & =Q_{\kappa }-S_{\kappa }{\left(S_{\kappa }R_{\kappa }^{-1}\right)}^{T}-S_{\kappa }R_{\kappa }^{-1}S_{\kappa }^{T}+S_{\kappa }R_{\kappa }^{-1}R_{\kappa }{\left(S_{\kappa }R_{\kappa }^{-1}\right)}^{T}\\ & =Q_{\kappa }-S_{\kappa }R_{\kappa }^{-1}S_{\kappa }^{T} \end{array}$$

Based on (13) and (34), we have established a new system model without correlated noise. The new one-step prediction can be obtained in the following manner:

(41) $$\begin{array}{cc} {\overset{⌢}{X}}_{\kappa +1|\kappa } & =E[X_{\kappa +1}|Z_{\kappa }]\\ & =E[F_{\kappa }^{*}X_{\kappa }+U_{\kappa }+W_{\kappa }^{*}|Z_{\kappa }]\\ & =F_{\kappa }^{*}{\overset{⌢}{X}}_{\kappa }+U_{\kappa } \end{array}$$

(42) $$\begin{array}{cc} P_{\kappa +1|\kappa } & =E[{\tilde{X}}_{\kappa +1|\kappa }{\tilde{X}}_{\kappa +1|\kappa }^{T}|Z_{\kappa }]\\ & =E[(F_{\kappa }^{*}{\tilde{X}}_{\kappa }+W_{\kappa }^{*}){\left(F_{\kappa }^{*}{\tilde{X}}_{\kappa }+W_{\kappa }^{*}\right)}^{T}|Z_{\kappa }]\\ & =E[F_{\kappa }^{*}{\tilde{X}}_{\kappa }{\tilde{X}}_{\kappa }^{T}{\left(F_{\kappa }^{*}\right)}^{T}|Z_{\kappa }]+E[W_{\kappa }^{*}{\left(W_{\kappa }^{*}\right)}^{T}|Z_{\kappa }]\\ & =F_{\kappa }^{*}P_{\kappa }{\left(F_{\kappa }^{*}\right)}^{T}+Q_{\kappa }^{*} \end{array}$$

The new estimation of the state can be expressed as

(43) $$\begin{array}{cc} {\overset{⌢}{X}}_{\kappa +1} & =E[X_{\kappa +1}|Z_{\kappa }]\\ & =E[F_{\kappa }^{*}X_{\kappa }+U_{\kappa }+W_{\kappa }^{*}|Z_{\kappa }]\\ & ={\overset{⌢}{X}}_{\kappa +1|\kappa }+K_{\kappa +1}^{*}(Z_{\kappa +1}-H_{\kappa +1}{\overset{⌢}{X}}_{\kappa +1|\kappa }) \end{array}$$

(44) $$\begin{array}{cc} P_{\kappa +1}= & E[{\tilde{X}}_{\kappa +1}{\tilde{X}}_{\kappa +1}^{T}|Z_{\kappa +1}]\\ & =E[(X_{\kappa +1}-{\overset{⌢}{X}}_{\kappa +1}){\left(X_{\kappa +1}-{\overset{⌢}{X}}_{\kappa +1}\right)}^{T}|Z_{\kappa +1}]\\ & =E[(X_{\kappa +1}-{\overset{⌢}{X}}_{\kappa +1|\kappa }-K_{\kappa +1}^{*}(Z_{\kappa +1}-H_{\kappa +1}{\overset{⌢}{X}}_{\kappa +1|\kappa })){\left(\cdot \right)}^{T}|Z_{\kappa +1}]\\ & =E[({\tilde{X}}_{\kappa +1|\kappa }-K_{\kappa +1}^{*}(H_{\kappa +1}{\tilde{X}}_{\kappa +1|\kappa }+V_{\kappa })){\left(\cdot \right)}^{T}|Z_{\kappa +1}]\\ & =E[((I-K_{\kappa +1}^{*}H_{\kappa +1}){\tilde{X}}_{\kappa +1|\kappa }+K_{\kappa +1}^{*}V_{\kappa }){\left(\cdot \right)}^{T}|Z_{\kappa +1}]\\ & =(I-K_{\kappa +1}^{*}H_{\kappa +1})E[{\tilde{X}}_{\kappa +1|\kappa }{\tilde{X}}_{T}^{\kappa +1|\kappa }|Z_{\kappa +1}]{\left(I-K_{\kappa +1}^{*}H_{\kappa +1}\right)}^{T}+K_{\kappa +1}^{*}E[V_{\kappa }V_{\kappa }^{T}|Z_{\kappa +1}]K_{\kappa +1}^{*T}\\ & =(I-K_{\kappa +1}^{*}H_{\kappa +1})P_{\kappa +1|\kappa }{\left(I-K_{\kappa +1}^{*}H_{\kappa +1}\right)}^{T}+K_{\kappa +1}^{*}R_{\kappa }K_{\kappa +1}^{*T}\\ & =P_{\kappa +1|\kappa }-K_{\kappa +1}^{*}H_{\kappa +1}P_{\kappa +1|\kappa } \end{array}$$

where the filter gain $K_{\kappa +1}^{*}$ has the same form as (31), but $K_{\kappa +1}^{*}$ is calculated based on (44):

(45) $$K_{\kappa +1}^{*}=P_{\kappa +1|\kappa }H_{\kappa +1}^{T}{\left(H_{\kappa +1}P_{\kappa +1|\kappa }H_{\kappa +1}^{T}+R_{\kappa +1}\right)}^{-1}$$

### 3.3. Design of ETCNF

Next, the ETCNF is formulated by integrating the event-triggered mechanism with correlation noise filtering. The one-step prediction is consistent with (41) and (42). And the new estimation of state can be rewritten as

(46) $${\overset{⌢}{X}}_{\kappa +1}={\overset{⌢}{X}}_{\kappa +1|\kappa }+\gamma_{\kappa +1}K_{\kappa +1}^{*}(Z_{\kappa +1}-H_{\kappa +1}{\overset{⌢}{X}}_{\kappa +1|\kappa })$$

(47) $$P_{\kappa +1}=P_{\kappa +1|\kappa }-\gamma_{\kappa +1}K_{\kappa +1}^{*}H_{\kappa +1}P_{\kappa +1|\kappa }$$

If $\gamma_{\kappa +1}=1$, the measurement information $Z_{\kappa +1}$ at time κ+1 has arrived, and it is not an outlier. So, the $Z_{\kappa +1}$ should be involved in the state estimation. If $\gamma_{\kappa +1}=0$, the $Z_{\kappa +1}$ should be an outlier, and we must isolate it through an event-triggered mechanism; thus, we can acquire the following:

(48) $${\overset{⌢}{X}}_{\kappa +1}={\overset{⌢}{X}}_{\kappa +1|\kappa }$$

(49) $$P_{\kappa +1}=P_{\kappa +1|\kappa }$$

**Figure 2:**
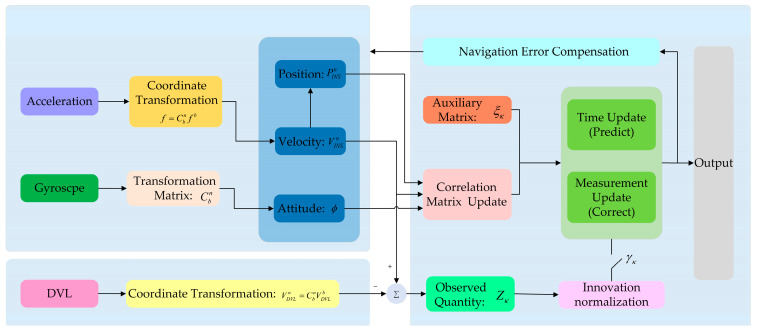
Diagram of the ETCNF algorithm of the INS/DVL integrated navigation system.

The overall structure of the ETCNF algorithm is shown in Figure 2. Different from the traditional loose integration method, the ETCNF method proposed in this paper de-correlates system noise and the observed noise by introducing an auxiliary matrix. At the same time, observation vectors are no longer unconditionally involved in measurement updates but filtered by an event-triggered mechanism.

## 4. Simulation Example

In this Section, due to limitations regarding the experimental conditions, a vehicle test was used to simulate underwater vehicles to illustrate the effectiveness of the proposed ETCNF algorithm. The angular velocity and acceleration data from the INS and the velocity from the GPS replace the velocity of DVL to simulate the underwater navigation environment (for the INS/DVL integrated navigation system, the velocity information from different sensors does not affect the algorithm test). In the meantime, we believe that in the same environmental background or medium, these two types of noise must be correlated, only with different degrees of correlation. When the INS and DVL are both in the underwater marine environment, both the process noise and the measurement noise will be affected by ocean reverberations, resulting in correlation. During the driving of the vehicle, the sensors used in our experiments are also affected by the same conditions in the atmosphere, resulting in a correlation between process noise and measurement noise. This correlation is different from that generated in the marine environment only in terms of the degree of correlation.

We demonstrate the effectiveness of the proposed algorithm by comparing the performances of the following algorithms:

- KF: The traditional INS and DVL loose combination is performed by Kalman filtering.
- ETKF: The traditional loose combination of INS and DVL is achieved by Kalman filtering on the basis of adding an event-triggered mechanism.
- CNF: The filter mentioned in this paper is processed by decorrelation through the introduction of an auxiliary matrix without an event-triggered mechanism.
- ETCNF: The algorithm proposed in this paper includes decorrelation filtering and an event-triggered mechanism.

In this experiment, the vehicle was selected and equipped with a data acquisition system. The INS uses HG4930 and sets the output frequency to 100 Hz. Its performance is shown in Table 1. The GPS receiver uses UM982 with an output frequency of 1 Hz.

**Figure 3:**
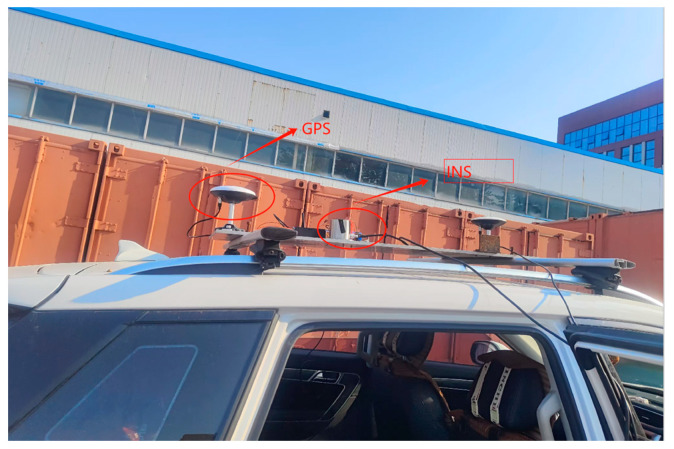
Vehicle test equipment.

The installation position of each sensor is shown in Figure 3. The INS and GPS antennas are rigidly connected to the vehicle.

**Figure 4:**
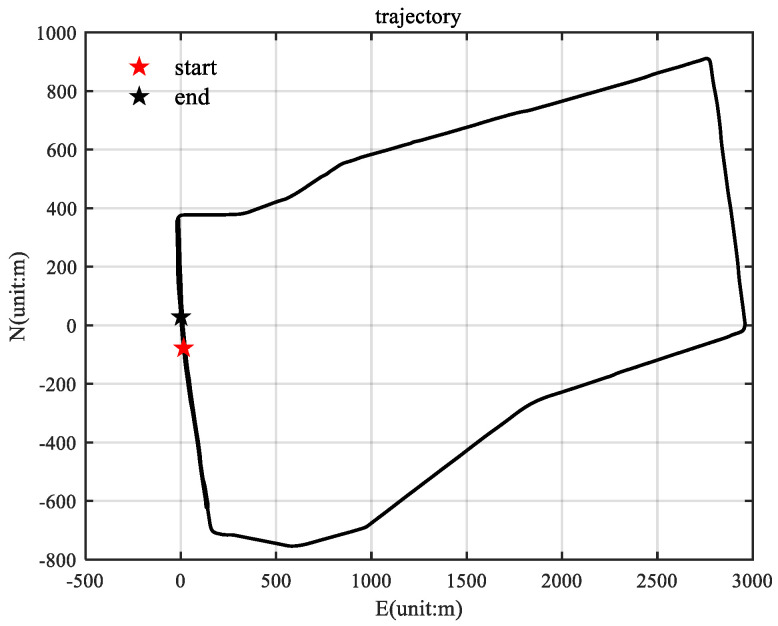
Vehicle trajectory diagram.

The running track used is shown in Figure 4, and the total sailing time was 1558.8 s. The whole test process was carried out under good GPS signal conditions, and it included deceleration, acceleration, steering, stopping, and other movements. In this experiment, the lever arm compensation for the GPS and INS was set as $\left[\begin{array}{ccc} -0.195 & -0.160 & 0.723 \end{array}\right]$ m (using the right–front–up coordinates). The reference truth values were calculated using the loose combination output of Inertial Explorer.

**Figure 5:**
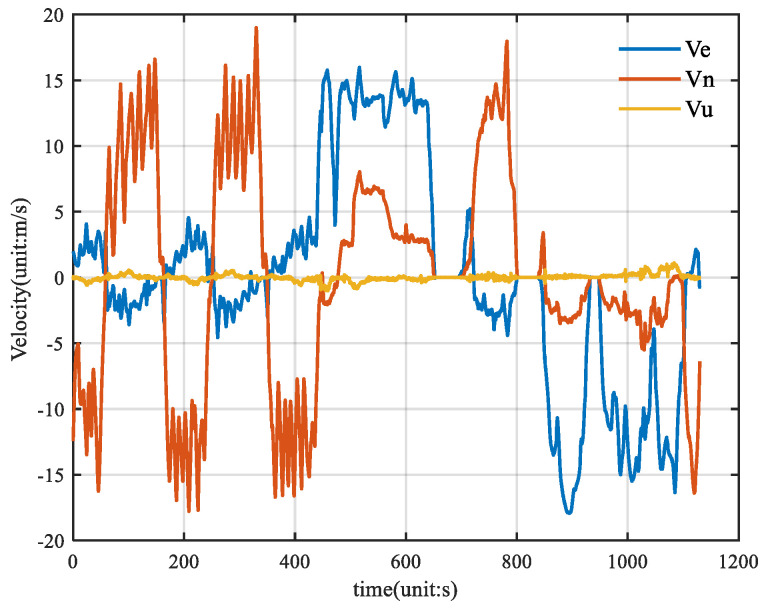
Vehicle velocity reference truth diagram.

**Figure 6:**
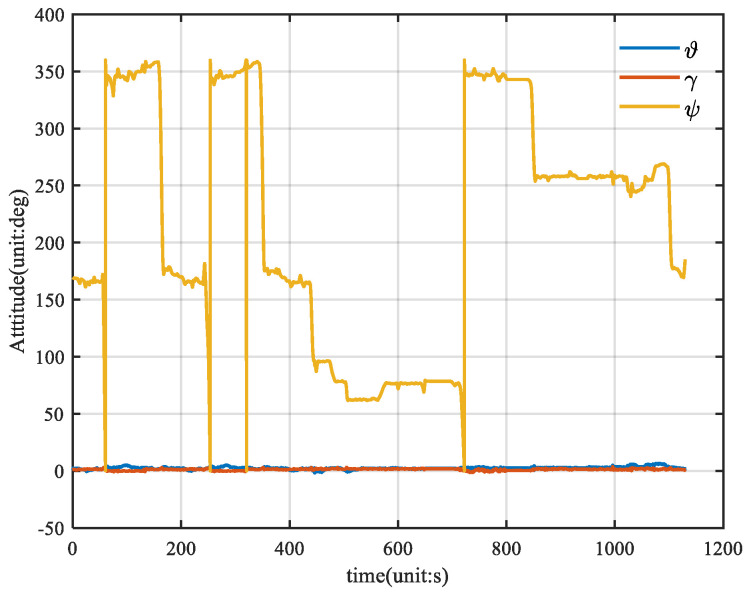
Vehicle attitude reference truth diagram.

Figure 5 shows the velocity of the vehicle, and Figure 6 shows the attitude of the vehicle, where θ is the pitch angle, γ is the roll angle, and ψ is the heading angle.

**Figure 7:**
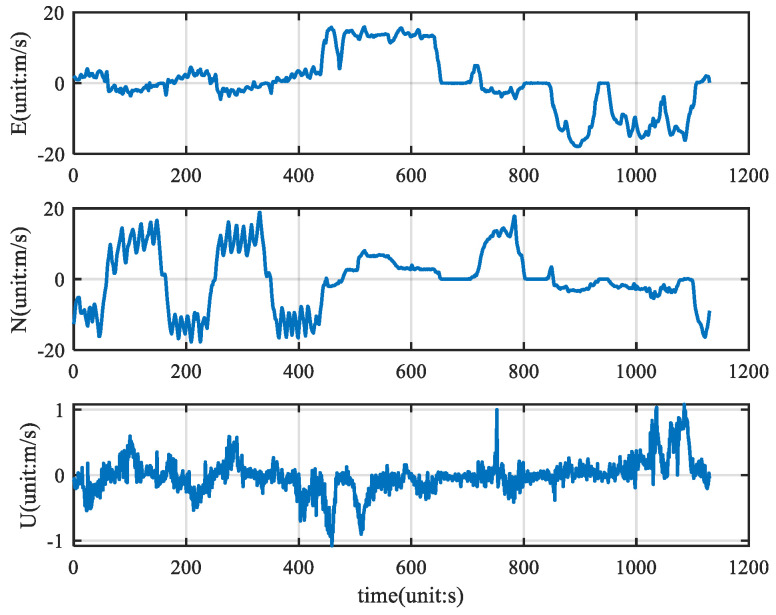
Velocity measurement.

The angular velocity, acceleration, and GPS velocity information collected by the vehicle is filtered by the KF, ETKF, CNF, and ETCNF respectively. The velocity information collected by the GPS is shown in Figure 7.

**Figure 8:**
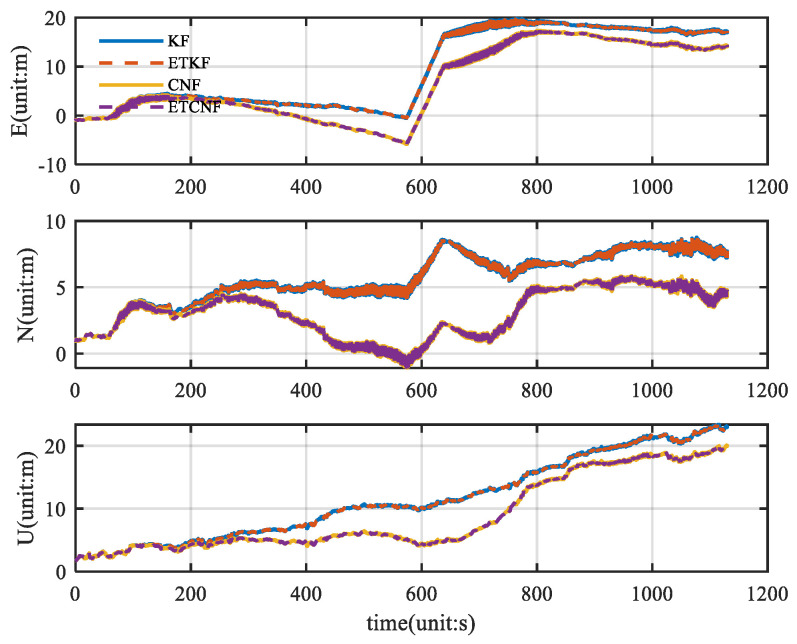
Comparison of position error for four methods without velocity measurement outliers.

As can be seen from Figure 7, there are almost no outlier values in the original velocity observations. Using good velocity observations, we compared the KF, ETKF, CNF, and ETCNF. The resulting position error is shown in Figure 8.

**Figure 9:**
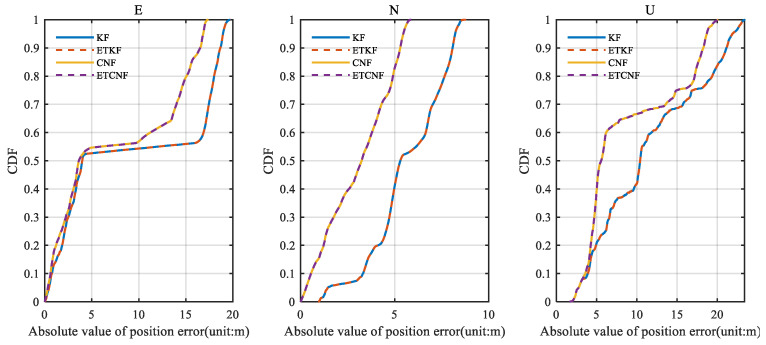
The CDF of position error for four methods without velocity measurement outliers.

Figure 8 illustrates the corresponding position error. Since there are no outliers in the original velocity observations, all the observations are integrated in the measurement update through the event-triggered mechanism. This results in KF position errors consistent with the ETKF and CNF results consistent with the ETCNF. It can be seen from the figure that although the position errors are all increasing, the proposed decorrelation algorithm has a smaller position error than the traditional algorithm. According to Figure 8, the absolute value of the position error was taken to be the horizontal axis, and the corresponding cumulative distribution function (CDF) graph was created, yielding Figure 9. The CDF can clearly display the distribution probability that is less than the absolute value of the corresponding position error. Figure 9 clearly shows that in the three directions of E-N-U, the CDF curves of the CNF and ETCNF algorithms are higher than those of the KF and ETKF algorithms, indicating that the position errors of the CNF and ETCNF algorithms are lower than those of the KF and ETKF algorithms, and the positioning accuracy is better. This is because the CNF algorithm effectively suppresses correlated noise.

To further illustrate the position error, the space position error formula was introduced:

(50) $$\Delta P=\sqrt{\Delta P_{E}^{2}+\Delta P_{N}^{2}+\Delta P_{U}^{2}}$$

**Figure 10:**
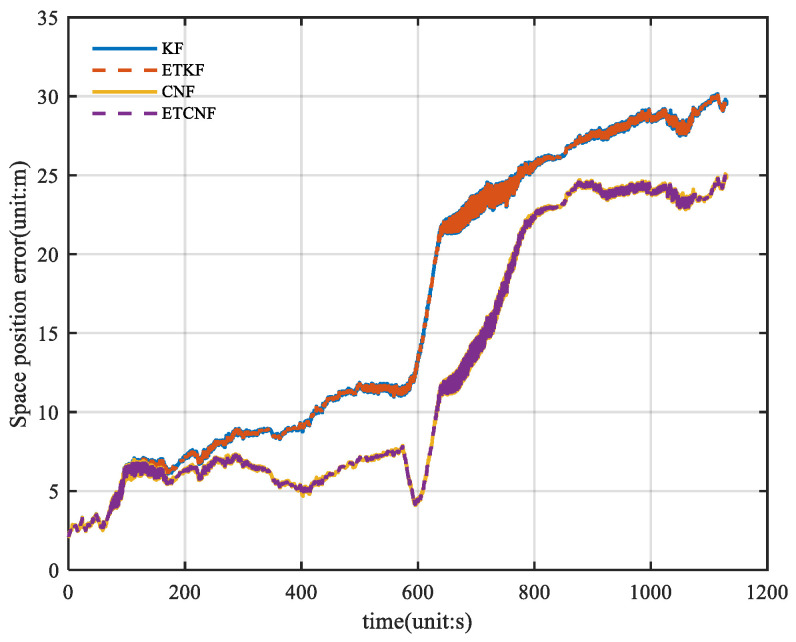
Curve of space position error for four methods without velocity measurement outliers.

**Figure 11:**
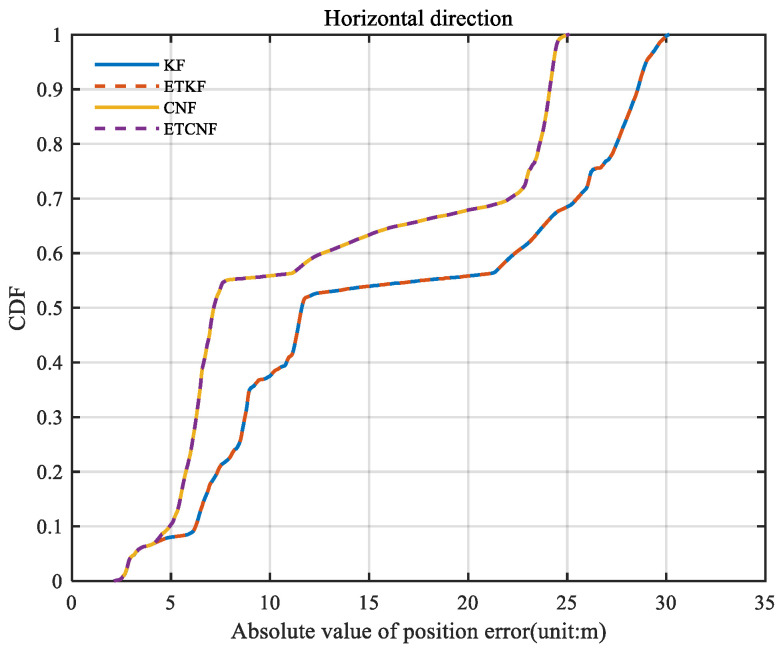
The CDF of curve of space position error without velocity measurement outliers.

Through Formula (50), the spatial position errors of the four algorithms were calculated, and the results are shown in Figure 10. The figure shows that the maximum value for KF and ETKF is 30.15 m, and the maximum value for CNF and ETCNF is 25.05 m. Compared with the loosely integrated method, the position accuracy of the proposed method is 16.2% better. Figure 11 shows the relevant CDF information from Figure 10. As can be seen in Figure 11, the position error distribution of the CNF and ETCNF algorithms is smaller than that of KF and ETKF algorithms, and the position error of the CNF and ETCNF algorithms is lower than that of the other two methods.

The RMSE values of the position errors of KF, ETKF, CNF, and ETCNF were calculated, and the formula is expressed as follows:

(51) $$RMSE=\sqrt{\frac{\sum_{i}^{N}{\left(x_{i}-{\bar{x}}_{i}\right)}^{2}}{N}}$$

**Figure 12:**
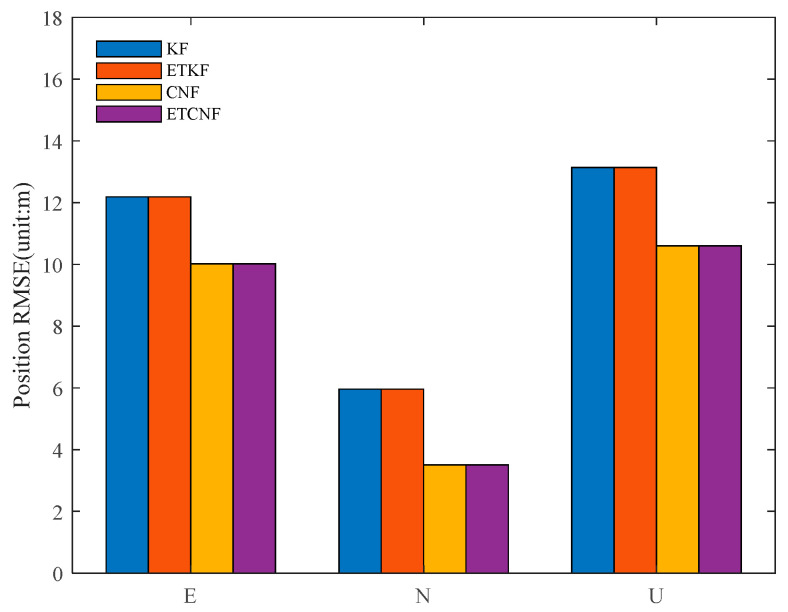
RMSEs for four methods without velocity measurement outliers.

The RMSEs of the four algorithms are shown in Figure 12, and their specific values are presented in Table 2. Compared with the KF-and-ETKF method, the proposed CNF-and-ETCNF method reduces the RMSE of position error in three directions of east, north, and up by 17.8%, 41.15%, and 19.3%, respectively. The positioning performance of the CNF algorithm is superior to that of KF.

**Figure 13:**
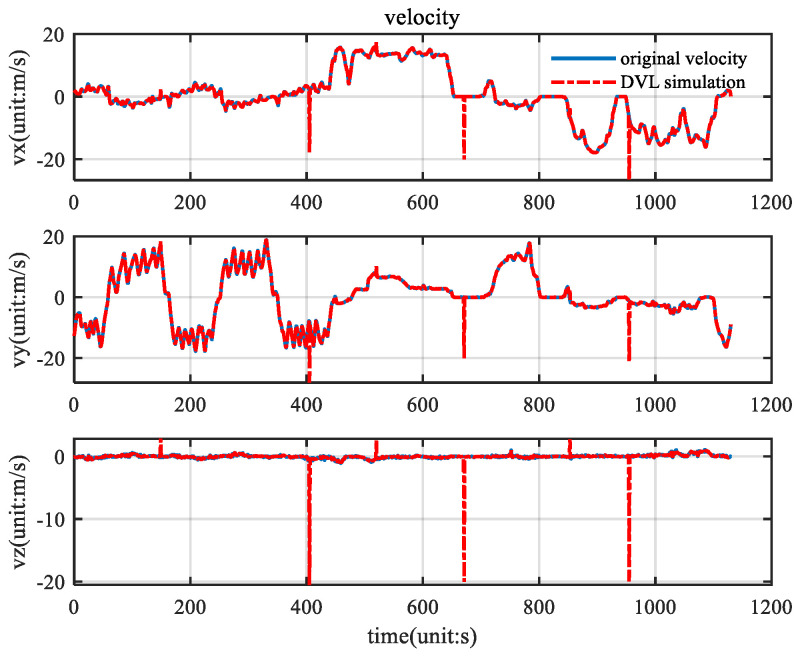
Velocity measurement with outliers.

In order to compare the positioning performance of CNF and ETCNF when outliers appear in the DVL, random outliers were added based on the velocity in Figure 3. By using the binomial distribution function, the occurrence probabilities of outliers were set to 0.01 and 0.005, and the values of the outliers were set to −20 m/s, 3 m/s, and 10 m/s. The specific velocity measurements are shown in Figure 13.

**Figure 14:**
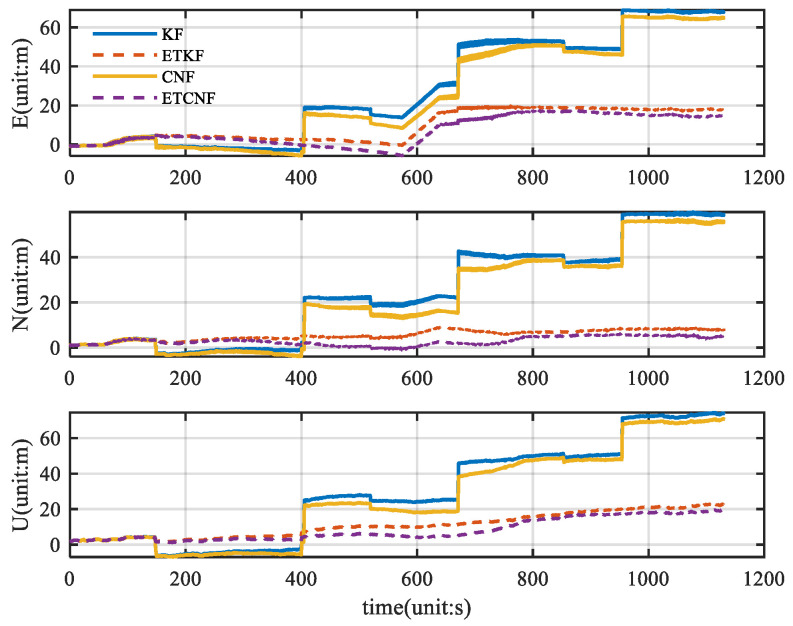
Comparison of position errors for four methods with velocity measurement outliers.

**Figure 15:**
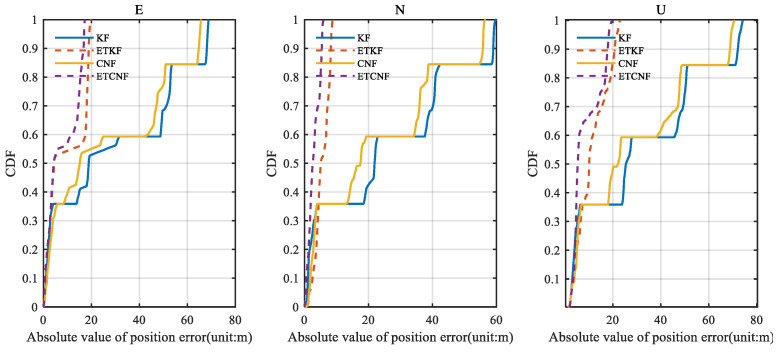
The CDF of position errors for four methods with velocity measurement outliers.

In Figure 14, the ETKF and ETCNF position error curves are more stable and coherent, while the KF and CNF position error curves change sharply. This is due to the error introduced by KF and CNF algorithms, where the observed outlier participates in state estimation. It is clear from Figure 15 that the ETKF and ETCNF position errors are smaller. This is precisely because the event-triggered- mechanism isolates the measurement outlier from the results of the measurement update. And compared with the ETKF and ETCNF algorithms, the ETCNF algorithm showed better performance in the three E-N-U directions. The position error of the CND algorithm is also slightly smaller than that of KF. The superiority of de-correlation filtering has thus been demonstrated.

**Figure 16:**
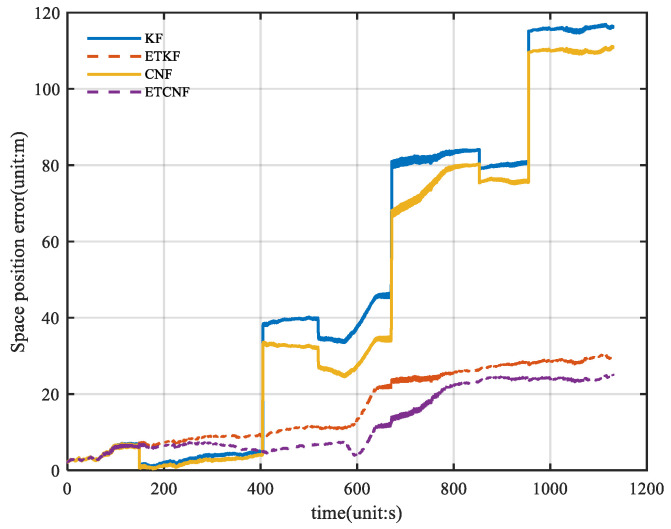
Curve of space position errors for four methods with velocity measurement outliers.

**Figure 17:**
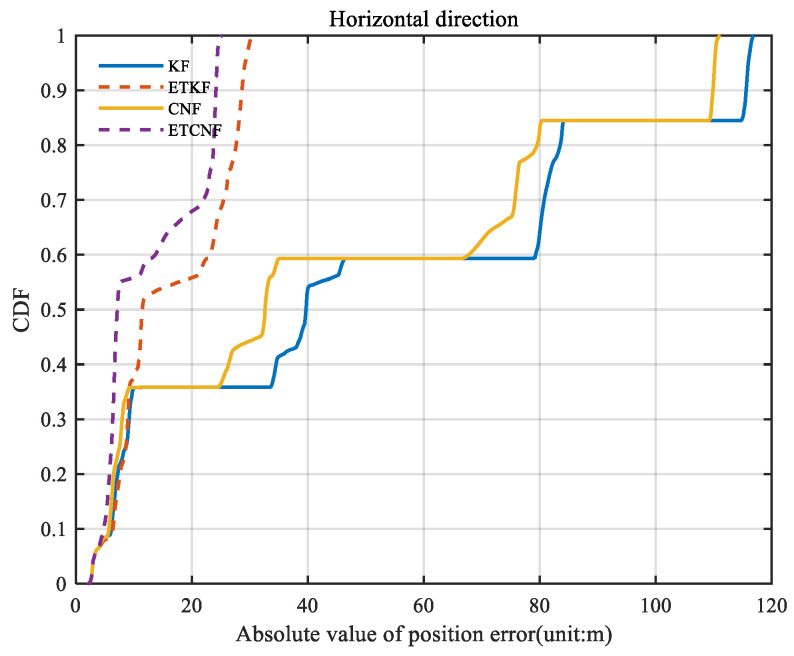
The CDF of curve of space position errors for four methods with velocity measurement outliers.

Figure 16 illustrates the spatial position errors of the four algorithms. The figure shows that the maximum values of the KF, ETKF, CNF, and ETCNF methods are 116.9 m, 30.36 m, 111.1 m, and 24.98 m, respectively. Compared with the KF method, the positioning accuracy of the proposed ETKF, CNF, and ETCNF methods improved by 74.03%, 5.22%, and 78.63%, respectively. Figure 17 shows the CDF curve for Figure 16. It can also be clearly seen from Figure 17 that the ETCNF algorithm has the best horizontal positioning accuracy, followed by the ETKF algorithm. The horizontal positioning performance of these two algorithms is far better than that of the CNF and KF algorithms.

**Figure 18:**
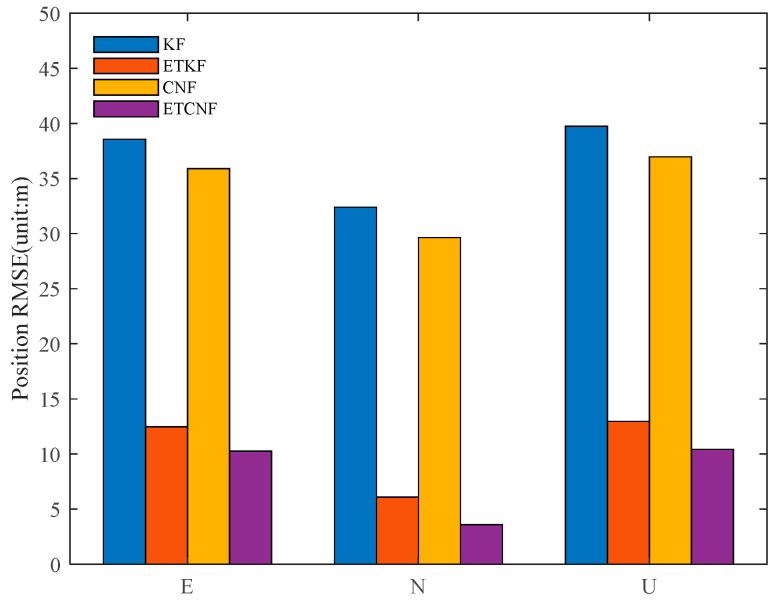
RMSE for four methods with velocity measurement outliers.

Figure 18 illustrates the positional RMSE values of the four methods in the presence of outliers, and their specific values are presented in Table 3. It can be seen from the table that compared with the KF method, the position-error RMSE for the ETKF algorithm is 67.67%, 81.25%, and 67.38% lower in the east, north, and upper directions, respectively. Compared with CNF, the position-error RMSE for the proposed ETCNF method in the east, north, and up directions is 71.46%, 87.94%, and 71.83% lower, respectively.

## 5. Conclusions

This paper introduces a novel INS/DVL filter named ETCNF. The standard Kalman filter suffers from inaccurate state prediction in the presence of correlated noise. The ETCNF algorithm resolves this issue by introducing an auxiliary matrix to decouple the correlated noise and establishing a new state equation for state estimation. Moreover, when outliers are detected in the DVL velocity observations, the ETCNF algorithm adopts an event-triggered mechanism. It uses normalized innovation to identify and eliminate these outliers. A simulation experiment was conducted in this study. By comparing the KF, ETKF, CNF, and ETCNF algorithms, we verified that the ETCNF algorithm can effectively improve the positioning performance of the INS/DVL integrated navigation system through the decoupling of correlated noise. Meanwhile, the event-triggered mechanism can effectively isolate velocity outliers, preventing errors caused by the inclusion of outliers in state estimation and significantly boosting the robustness of the system.

## Figures and Tables

**Table 1:** INS error parameter.

INS Errors	Value
Gyro bias standard deviation	15 (°/h)
Accelerometer bias standard deviation	150 (mGal)
Velocity random walk	$0.2\;(m/s/\sqrt{h}$)
Angle random walk	$0.2\;({}^{\circ}/\sqrt{h}$)

**Table 2:** RMSE values for position error without velocity measurement outliers.

Method	East	North	Up
KF	12.19	5.9634	13.142
ETKF	12.19	5.9634	13.142
CNF	10.02	3.5094	10.606
ETCNF	10.02	3.5094	10.606

**Table 3:** RMSE values for position error with velocity measurement outliers.

Method	East	North	Up
KF	38.570	32.397	39.749
ETKF	12.469	6.0756	12.965
CNF	35.908	29.65	36.977
ETCNF	10.248	3.5765	10.416

## Data Availability

Data are only available on request due to privacy or ethical restrictions. The data presented in this study are available on request from the corresponding author.
